# ICG Lymphography Confirms the Presence of an Alternative Lymph Drainage Pathway Following Long-Term Manual Therapy: A Case for Preserving Traditional MLD Approaches

**DOI:** 10.3390/reports8020063

**Published:** 2025-05-06

**Authors:** Mary Wakefield, Jan Douglass, Diane Lacey, Neil Piller, Linda Blanchfield

**Affiliations:** 1 Dove Hospice & Wellness, Auckland 1071, New Zealand; mary.wakefield@dovehospice.org.nz; 2 College of Medicine and Dentistry, James Cook University, Douglas, QL 4811, Australia; 3 Lymph Therapies, Auckland 2120, New Zealand; 4 College of Medicine and Public Health, Flinders University South Australia, Bedford Park, SA 5042, Australia; 5 Dr. Vodder School International, Vancouver, BC V7J 2P7, Canada

**Keywords:** alternative lymphatic pathways, breast cancer-related lymphedema (BCRL), indocyanine green (ICG) lymphography, lymphedema management, manual lymphatic drainage (MLD)

## Abstract

**Background and Clinical Significance**: Breast cancer-related lymphedema (BCRL) is a chronic condition affecting up to 20% of breast cancer survivors. Manual lymphatic drainage (MLD) has traditionally included techniques to redirect lymph flow toward alternative pathways when axillary drainage is impaired. However, emerging imaging techniques suggest that most lymph continues to drain toward the ipsilateral axilla, and this has led to the widespread uptake of treatment protocols that exclude traditional redirecting movements, even in cases where personalized imaging is unavailable. **Case Presentation**: This case report describes a woman with BCRL affecting the right arm and hand who underwent 3 years of conservative lymphedema therapy, including MLD and self-massage techniques that incorporated traditional redirection strategies. Pre-operative indocyanine green (ICG) lymphography, performed after prolonged conservative treatment, confirmed the presence of an open alternative drainage pathway bypassing the axilla and demonstrated dermal flow along the redirected pathways towards a previously described “radial” pathway. These findings suggest that targeted manual therapy may have reinforced or optimized this compensatory route. **Conclusions**: This case highlights the potential risk of relying on a single form of assessment and generalized cohort imaging studies to guide individualized MLD protocols. In the absence of personal imaging, prematurely abandoning traditional redirection techniques may limit opportunities to establish functional alternative pathways, particularly in early edema in patients who have this anatomical variation. ICG lymphography provides valuable insight into compensatory lymphatic drainage. However, until imaging protocols are standardized and individual imaging is widely accessible, retaining traditional MLD techniques for newly diagnosed BCRL may be crucial for optimizing treatment outcomes. Future research should explore the long-term impact of manual therapy on alternative pathway development and function.

## 1. Introduction and Clinical Significance

Lymphedema is a well-recognized consequence of breast cancer treatment, commonly arising after mastectomy, lymph node dissection, chemotherapy, and/or radiotherapy. It results from disrupted lymphatic drainage, leading to the accumulation of protein-rich fluid and progressive tissue changes. According to the International Society of Lymphology [1], the clinical progression of lymphedema occurs across three stages, preceded by a latent (Stage 0) phase in which subclinical fluid accumulation and tissue changes develop before visible swelling occurs. During this stage, patients may experience transient or persistent symptoms for months or even years before overt signs emerge. Treatment for lymphedema is broadly categorized into conservative (non-operative) and surgical approaches, with conservative management historically forming the foundation of best-practice care.

In Australia alone, an estimated 57,500 to 90,000 people (total population 27 million) are living with oncology-related secondary lymphedema, with no single database capturing all cases [2,3]. In New Zealand, breast cancer remains a leading malignancy among women. In 2022, 3440 women were diagnosed with breast cancer, representing 27% of all female cancer cases, and 38 men were diagnosed, accounting for just over 1% of all breast cancer diagnoses [4]. Approximately 20% of breast cancer survivors will develop arm lymphedema, suggesting that hundreds of new cases of secondary lymphedema will emerge annually in New Zealand alone among a total population of only 5.2 million, further emphasizing the ongoing burden of this condition.

For over half a century, the standard approach to breast cancer-related lymphedema (BCRL) has been a two-phase multimodal program. The first phase involves an intensive protocol administered by trained allied health professionals, incorporating manual lymphatic drainage (MLD), multi-layer compression bandaging, exercise, and skin-care to reduce swelling and optimize skin integrity. The second phase—maintenance—shifts responsibility to the patient, focusing on self-care strategies such as self-massage, compression garment use, exercise, and continued skin-care [1].

Despite the lymphatic system’s capacity for spontaneous recovery, approximately one in five breast cancer survivors will develop arm lymphedema within their lifetime, with most cases emerging within 18 months of cancer treatment [5]. The lifelong consequences—including reduced mobility, discomfort, recurrent infections, and diminished quality of life—are well-documented [6].

One of the challenges in BCRL management is the highly individualized nature of lymphatic anatomy. While the majority of individuals exhibit a drainage pattern where the skin of the hand and arm drains toward the superficial axillary lymph nodes, approximately 40% possess an alternative pathway in which lymph from the radial hand is directed into deeper vessels following the cephalic vein toward the supra- and subclavicular lymph nodes [7]. This pathway often remains intact after breast cancer treatment, leading some researchers to hypothesize that its presence may reduce the risk of developing lymphedema [8]. Although not universally present, long-standing MLD protocols have traditionally incorporated techniques designed to redirect fluid toward this alternative pathway, maximizing the body’s natural compensatory capacity [9]. However, further detailed imaging studies are needed to confirm the prevalence and function of these routes in lymphedema-affected individuals.

Recent advances in surgical interventions, such as lympho-venous anastomosis (LVA), have driven the development of improved lymphatic imaging techniques. The use of indocyanine green (ICG) fluorescence lymphography has provided significant insights into post-treatment lymphatic drainage patterns. Emerging findings indicate that, despite axillary lymph node disruption, the majority of lymph from the upper limb continues to drain toward the ipsilateral axilla, particularly in mild cases [10]. These results have been extrapolated to the broader BCRL population and have influenced modifications in clinical practice at all stages of lymphedema, omitting traditional redirection techniques toward alternative pathways. This shift in clinical practice is occurring even when individual imaging is unavailable to confirm whether such an approach is appropriate for a given patient, and does not follow the recommendation of the researchers [10].

This case report is on a woman who developed BCRL in her arm and hand. Pre-operative ICG lymphograms which become available after a period of conservative therapy appear to support the treatment approach, which had followed traditional MLD principles to redirect fluid around the ipsilateral axilla toward the lateral arm and supraclavicular lymph node region (Supplementary Figure S1). Both professional and self-massage lymphatic drainage techniques had promoted fluid movement toward this pathway, and post-treatment imaging revealed that, while some lymph fluid drained through the axillary pathway, a significant portion of dermal fluid followed the redirected pathways. This raises a crucial question: would the same compensatory drainage patterns have been observed if the therapist had followed cohort-based data to exclusively direct lymph flow toward the ipsilateral axilla? To our knowledge, there are no studies comparing traditional redirecting protocols with newer recommendations for ipsilateral drainage. 

Clinical Significance: This case underscores the potential risk in basing individualized treatment plans on generalized cohort imaging studies alone. In the absence of patient-specific imaging, the premature abandonment of traditional MLD techniques may result in missed opportunities to establish and reinforce alternative lymphatic pathways, potentially compromising long-term management outcomes.

## 2. Case Presentation

### 2.1. Case Description

The 47-year-old female patient was diagnosed with Stage 2 breast cancer following a routine mammogram in 2011 and underwent a right mastectomy with 10 lymph nodes removed from the axilla. An immediate expander/implant reconstruction was performed, followed by chemotherapy (Fluorouracil Epirubicin hydrochloride and Cyclophosphamide for three cycles and Taxol given weekly for 9 weeks), with radiotherapy to the right chest wall (50 Gy in 25 fractions). The patient was active with a stable BMI of 24.1 (height 171 cm and weight 70.4 kg).

### 2.2. Patient History

Over the following five years, the patient was hospitalized three times due to cellulitis originating in her proximal right forearm. By early 2019, she began noticing swelling over the posterior lateral elbow and proximal forearm which she described as “setting up home”. She sought professional assistance from a private physiotherapist who treated her on three occasions for “elbow strain” using MLD and a four-chamber arm sequential pump (Bio Compression Systems, Moonachie, NJ, USA). Arm measurements were not taken and the patient ceased treatment as she did not believe it was an “elbow strain”. Following a GP referral, the patient was seen in May 2019 at a public hospital outpatient lymphedema clinic where twenty days of cohesive multi-layer bandaging was used without MLD. The patient attended weekly to re-measure, with skin assessment and rebandaging. She reflected that her arm felt uncomfortable during this time and began to notice swelling in her hand which had not previously been an issue. The patient felt “unheard” when she verbalized her distress regarding this new development, something that deeply frustrated her, and she subsequently self-referred to Dove Hospice and Wellness, Auckland, for lymphedema management, where care was shared with the outpatient lymphedema clinic. On presentation in August 2019, a 5 second pressure test showed pitting edema over the entire forearm and to a lesser degree in the dorsum of the hand, where the metacarpal bones were still visible.

### 2.3. Treatment Approach

**Figure 1:**
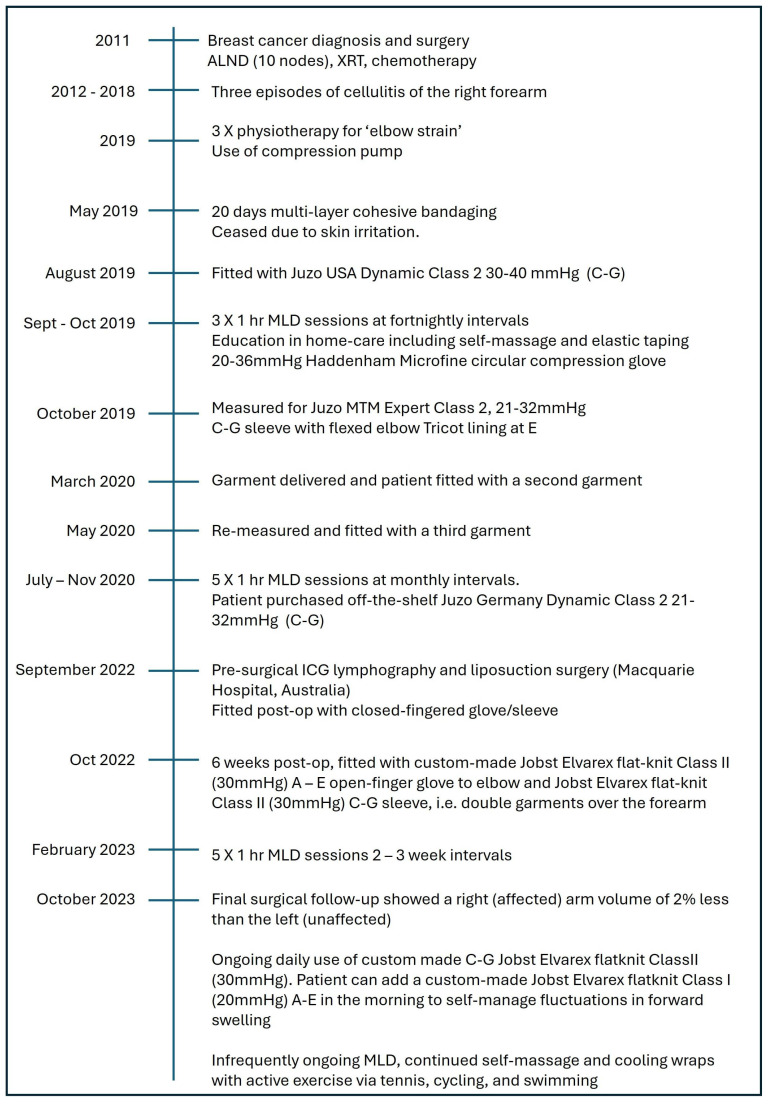
Timeline of breast cancer-related lymphedema diagnosis and treatment. MLD protocol followed redirection protocols according to Vodder. ALND = axillary lymph node dissection; MLD = manual lymph drainage; ICG = indocyanine green.

A long-term treatment plan was initiated using MLD techniques, according to Vodder [8], and regular shoulder mobilization techniques. MLD treatment protocols have traditionally aimed to redirect dermal fluid from the ipsilateral thoracic wall toward the parasternal and contralateral lymph nodes and from the upper limb toward the radial pathway, and this protocol was followed both during the MLD treatments and in the self-massage taught to the patient. MLD was performed eight times over 14 months, and adjunctive elastic therapeutic taping was used on three occasions to address hand swelling (Figure 1).

**Figure 2:**
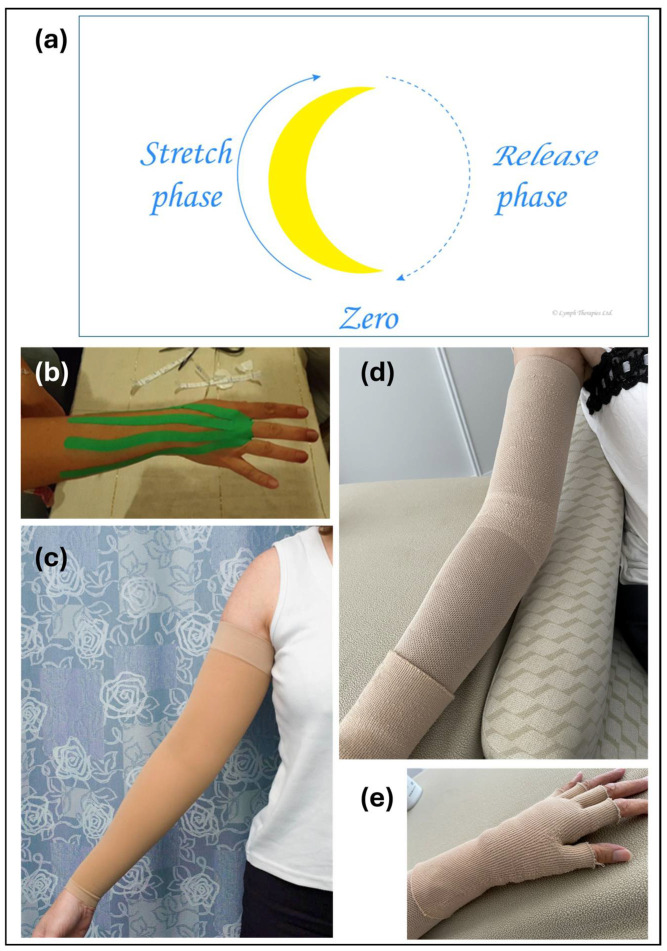
(**a**) A diagram representing Dr Vodder stationary circles showing the increasing and then decreasing stretch and release phases moving the skin in a circular path to create multidirectional shearing forces in the underlying tissue. Image created by Diane Lacey © 2022 and reproduced with permission. (**b**) An example of the hand-taping technique. (**c**) An example of a Jobst Elvarex flat-knit Class I (20 mmHg) C-G round knit, an off-the-shelf arm sleeve; (**d**) an example of a custom-made Jobst Elvarex flat-knit Class II (30 mmHg) C-G arm sleeve; (**e**) an example of a Jobst Elvarex flat-knit Class I (20 mmHg) A–E fingerless glove. Images (band **c** by Jan Douglass, images (**d**) and (**e**) by Mary Wakefield.

Each of the MLD treatments followed the principles described by Vodder, which employ circular movement of the skin to transfer stretching and shearing forces to the underlying tissue to increase lymph formation and promote lymph flow (Figure 2a). Pressures are adapted according to the presentation, with stronger pressures applied to soften fibrotic areas. The self-massage protocol employed lymphatic effleurage by the patient, in this case, flat-handed stroking movements along the skin to encourage fluid movement and lymph flow. Despite differences in technique, the common intention is to move both trapped dermal fluid and lymph already within patent lymph vessels towards open (unaffected) contralateral lymph vessels and ipsilateral alternative drainage pathways. The elastic taping treatment involved strips of tape which were applied fanning over the palmar and dorsal skin of the hand and proximal forearm and extending toward the lateral aspect of the upper arm following the same pathway as the MLD and self-massage protocols to reinforce the dorsolateral pathway (Figure 2b). The patient was taught the same self-taping protocol and became proficient at it.

The patient was fitted temporarily with an off-the-shelf Class II circular knit compression sleeve, and although measurements were taken in October 2019 for a custom-made flat-knit sleeve (Juzo, Aichach, Germany), due to the global pandemic, this garment was not supplied until March 2020. A second sleeve was supplied at the same time, and in May 2020, a third garment was supplied and a strip of fibrosis padding (Mobiderm, Thuasne, France) was added along the posterior forearm under the compression sleeve. A 20–36 mmHg Microfine compression glove (Haddenham Healthcare, Long Crendon, Buckinghamshire, UK) was prescribed, and the patient was educated on the use of the glove and the application of the lymph tapes as needed to manage her hand swelling. Figure 2 shows examples of the (c) round-knit, (d) flat-knit, and (e) fingerless glove compression garments. Other aspects of lymphedema home-care were taught, including self-lymphatic drainage, and the patient frequently wrapped cold wheat bags around the forearm to manage symptoms. In September 2020, the patient purchased an off-the-shelf sleeve as she could not afford to replace the custom-made garments (Juzo Dynamic Max).

### 2.4. Lifestyle Integration

A highly active individual, the patient maintained a regular exercise regimen throughout her treatment and recovery. She participated in interclub tennis, swimming (initially daily, later reduced to 3–4 times a week before COVID-19, and then stopping during COVID-19 lockdown), and cycling, completing a 40 km round trip to attend lymphedema treatment appointments. She also worked part-time in a sports-related office position, which kept her active in her community.

### 2.5. Liposuction Assessment and Outcome

In mid-2022, the patient was assessed for a liposuction procedure to reduce excess lymphedema-related tissue in her right arm. The assessment included clinical examination by a surgical consultant, ICG lymphography, and compression garment evaluation with a specialist physiotherapist (Figure 2). The pre-operative measures showed a 44% greater volume in the right arm compared to the left. Liposuction was performed in September 2022, and post-operative follow-up showed a greater than 100% reduction in excess right arm volume. At a 2024 follow-up, the right arm remained 2% smaller than the left.

### 2.6. Ongoing Therapy and Self-Management

The patient continues to wear compression garments and utilize self-care strategies, maintaining functional capacity in her arm, including the daily use of a custom-made C-G Jobst Elvarex flat-knit Class II (30 mmHg) compression sleeve. The patient can add a custom-made Jobst Elvarex flat-knit Class I (20 mmHg) compression glove (A–E) in the morning to self-manage fluctuations in forearm swelling. She has infrequent ongoing MLD, continues to self-massage, and uses cooling wraps, with active exercise namely tennis, cycling, and swimming (Figure 1).

**Figure 3:**
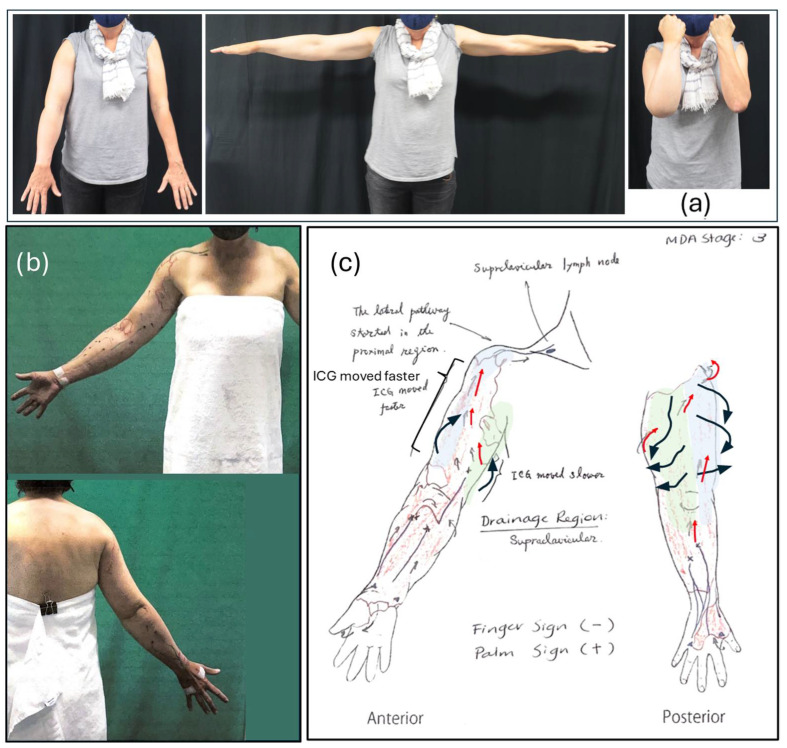
Pre-operative imaging showing (**a**) arm swelling; (**b**) pre-surgical mark-up, with blue lines indicating working lymphatic vessels and red crosses indicating visualized obstruction of lymphatic vessel; (**c**) hand-drawn anterior and posterior views of arm showing areas of dermal back flow (red shaded) and lymph drainage pathways as detected by ICG lymphography (red arrows), with overlay added to show normal lymphatic territories (blue and green shaded areas) and direction of upper arm lymph drainage (black arrows).

An analysis of the pre-operative ICG imaging showed a clear lymph pathway from the dorsolateral region of the upper arm, with a large patent radial vessel emptying into the subclavian pathways (Figure 3b). The original ICG images and report can be viewed in Supplementary Figure S1.

### 2.7. Subjective Results

When asked how the burden of lymphedema impacted on her life, the patient reported that it had “cost her freedom and independence”. It reduced her social activities, in particular limiting how much and how often she could play tennis, which also impacted on her mental health. She felt “down” that she could not join in and do things as she used to, and she constantly worried about getting another bout of cellulitis. The change after liposuction surgery was transformative: “I got back my old life, my old enjoyment in physical exercise and travel, my independence and confidence. I could lead an active life without being tied to hospitals and physical treatments”.

## 3. Discussion

The role of manual lymphatic drainage (MLD) as a cornerstone of lymphedema management has been increasingly scrutinized, with recent studies questioning its long-term efficacy. However, much of this research is based on short-term studies, and the long-term consequences of altering best-practice MLD therapy, particularly any potential risks to the patient, remain largely unknown. This case study highlights the critical importance of long-term therapy in establishing and reinforcing alternative lymphatic pathways, cautioning against a popular shift toward directing lymph flow exclusively to the ipsilateral axilla without individualized imaging.

The introduction of indocyanine green (ICG) lymphography has provided valuable insights into compensatory lymphatic drainage pathways, which are essential for refining both conservative and surgical interventions. In this case, ICG lymphography confirmed the presence of an accessory lymphatic pathway from the hand that bypassed the axillary lymph nodes and drained into deep nodes near the subclavian vein. The large and patent appearance of the radial lymph vessel suggests that the lymphatic system can adapt to increased demand, particularly when MLD and self-massage techniques are used to reinforce alternative drainage routes. This finding supports prior research suggesting that MLD actively contributes to the redirection and enhancement of compensatory lymphatic pathways [11]. A 2022 retrospective study on lymphoscintigraphy images from 80 primary (*n* = 44) or secondary (*n* = 36) patients with lower limb lymphedema showed that MLD increased the number of opened lymphatic pathways in 75% of the patients after only 15 min of MLD [9].

Multiple studies utilizing ICG lymphography have demonstrated that compensatory drainage develops in response to lymphatic obstruction. A retrospective analysis of 339 patients with secondary cancer-related lymphedema by Koelmeyer and Thompson [10] found that, in cases of upper limb lymphedema, the ipsilateral axilla remained the primary drainage region in 74.9% of patients, followed by the clavicular (41.8%) and parasternal (11.3%) pathways. In patients with mild lymphedema, 94.4% still drained to the ipsilateral axilla, suggesting that early intervention and redirection techniques may help preserve axillary drainage capacity in less severe cases. However, these cohort-based findings do not negate the potential for alternative pathways to be important drainage pathways in individual cases. One randomized controlled trial, which compared ICG-guided MLD to what was termed “traditional MLD”, had the potential to answer this question. However, the practitioners performing the “traditional MLD” were required to follow normal anatomical pathways without redirecting fluid around the axilla, and without adapting technique or pressure to the presenting skin and tissue pathology [12,13], both of which are generally considered key elements of “traditional” MLD protocols. Furthermore, since there were no significant differences between the groups, the results simply show that fluoroscopy-guided lymphatic drainage is not more effective than MLD performed incorrectly and therefore fails to enlighten us on whether correctly performed traditional MLD protocols should be changed when individual imaging is not available.

Two key questions remain unanswered by our report. Firstly, to what extent did the MLD protocol, as used by the therapist during the MLD sessions and reinforced by the patient during self-massage, contribute to the development of the radial/dorsolateral pathway? And secondly, is the capacity of traditional MLD practices to prevent subclinical (Stage 0) lymphedema from progressing to clinical disease underestimated? The patient almost certainly had a pre-existing radial drainage route, as evidenced by the absence of early hand swelling, but the extent to which MLD increased the capacity and function of these vessels remains uncertain, and it cannot be determined within this case report whether these pathways are the result of the MLD treatment or the patient’s natural anatomical variation. However, research suggests that inadequate ICG injection points in mapping studies may fail to detect posterior and dorsal pathways, leading to an underestimation of viable drainage routes [14]. If this leads to treatment strategies that do not include reinforcing these pathways, therapy effectiveness may be limited. While ICG fluoroscopy may be sufficient to guide surgical procedures such as liposuction and LVA, they may not give a complete picture of lymphedema status and should be considered alongside more traditional measures such as circumference (volume), segmental fluid loads (bio-impedance spectroscopy), and tissue stiffness (palpation) to guide therapeutic adaptations in technique [15].

Regarding the role of traditional MLD and self-massage protocols in the prevention of clinical signs of edema, when the patient was misdiagnosed with elbow strain could reinforcing the pre-existing radial pathway using MLD at the first sign of swelling, have prevented progression to frank lymphedema? Given that the lateral and dorsal pathways likely existed in a small or underdeveloped form before the patient’s breast cancer surgery, the absence of pre-surgical imaging means that we cannot determine whether these pathways would have spontaneously expanded without intervention. However, the fact that they were clearly functional and patent once imaging was performed suggests that MLD and self-massage may have played an important role in maintaining their viability and contributed to their increased capacity. Koelmeyer et al. state that their team has “repeatedly observed in our ICG lymphography clinic that the lymphedema affected limb appears to have developed compensatory lymphatic drainage, which can be augmented but not modified by MLD” [10]. This underscores the potential risk of abandoning traditional MLD techniques in favor of a standardized axillary-directed approach, especially for patients without individualized imaging to confirm their optimal drainage patterns.

Despite the valuable insights from this case, several limitations must be acknowledged. The absence of baseline ICG imaging prior to the application of conservative therapy makes it difficult to determine whether the observed pathways were pre-existing or therapy-induced, and the lack of other pre- and post-assessments, such as arm volume calculations, limits the ability to precisely quantify the direct impact of MLD on pathway development.

Future research should prioritize prospective longitudinal studies incorporating pre- and post-treatment ICG lymphography to better understand how MLD and self-massage influence lymphatic compensation. Standardizing ICG injection protocols to ensure the comprehensive mapping of posterior, dorsal, and lateral pathways will be crucial in refining both diagnostic and therapeutic strategies for lymphedema management.

## 4. Conclusions

This case reinforces the critical role of long-term lymphedema therapy—including MLD, compression therapy, and self-care strategies—in maintaining and enhancing alternative lymphatic drainage pathways in patients with arm lymphedema following breast cancer treatment. Notably, the imaging that was performed in preparation for a surgical procedure confirmed and supported the pathways that the therapist had been using in manual therapy, highlighting a rare and powerful instance of clinical intuition aligning with visual evidence. This underscores the potential for the skilled application of MLD to effectively engage and reinforce compensatory lymphatic routes even in the absence of imaging. To our knowledge, this is the first published report to describe this specific alignment between therapeutic practice and imaging-confirmed lymphatic architecture, marking a novel contribution to the literature. While emerging evidence highlights the ipsilateral axilla as a primary drainage site in many patients, a one-size-fits-all approach risks overlooking viable alternative pathways that could be critical for long-term lymphedema management.

The integration of ICG lymphography offers significant potential in identifying and optimizing compensatory pathways, emphasizing the need for individualized MLD protocols tailored to a patient’s specific lymphatic architecture. Until imaging becomes more widely accessible, the continued use of traditional techniques that support alternative pathways remains vital. This case underscores the importance of a multidisciplinary approach and calls for further prospective studies to refine treatment strategies, ensuring that MLD remains an evidence-based and personalized intervention for lymphedema care.

## Data Availability

The original contributions presented in this study are included in the article. Further inquiries can be directed to the corresponding author.

## Supplementary Materials

The following supporting information can be downloaded at: https://www.mdpi.com/article/10.3390/reports8020063/s1, Figure S1: ICG lymphography images and report.

## Abbreviations

The following abbreviations are used in this manuscript:

BCRL	Breast cancer-related lymphedema
MLD	Manual lymph drainage
ICG	Indocyanine green
