# Correction: Wakefield et al. ICG Lymphography Confirms the Presence of an Alternative Lymph Drainage Pathway Following Long-Term Manual Therapy: A Case for Preserving Traditional MLD Approaches. *Reports* 2025, *8*, 63

**DOI:** 10.3390/reports9010042

**Published:** 2026-01-29

**Authors:** Mary Wakefield, Jan Douglass, Diane Lacey, Neil Piller, Linda Blanchfield

**Affiliations:** 1 Dove Hospice & Wellness, Auckland 1071, New Zealand; mary.wakefield@dovehospice.org.nz; 2 College of Medicine and Dentistry, James Cook University, Douglas, QL 4811, Australia; 3 Lymph Therapies, Auckland 2120, New Zealand; diane@lymphtherapies.co.nz; 4 College of Medicine and Public Health, Flinders University South Australia, Bedford Park, SA 5042, Australia; neil.piller@flinders.edu.au; 5 Dr. Vodder School International, Vancouver, BC V7J 2P7, Canada; kobmld@gmail.com

The authors wish to make the below corrections to this paper [1].

## Error in Figure

In the original publication, there was a mistake in Figure 1 as published. Figure 1 has been updated with the correct name of the hospital and the corrected Figure 1 appears below. 

In the original publication, there was a mistake in Figure 3 as published and the legend for Figure 3c described alterations to the original image and the attribution for the origin of the images was missing. The image used in Figure 3c was altered with an overlay and was not correctly attributed to the institution that produced it. Figure 3c has been replaced with the original unaltered image and the corrected Figure 3c and it’s legend including the attribution appears below.

## Text Correction

There was an error in the original publication. The “Supplementary Materials” part should be deleted. Section 1, paragraph 7 refers to Supplementary Material. The supplementary images should be removed and not replaced:

This case report is on a woman who developed BCRL in her arm and hand. Pre-operative ICG lymphograms which become available after a period of conservative therapy appear to support the treatment approach, which had followed traditional MLD principles to redirect fluid around the ipsilateral axilla toward the lateral arm and supraclavicular lymph node region. Both professional and self-massage lymphatic drainage techniques had promoted fluid movement toward this pathway, and post-treatment imaging revealed that, while some lymph fluid drained through the axillary pathway, a significant portion of dermal fluid followed the redirected pathways. This raises a crucial question: would the same compensatory drainage patterns have been observed if the therapist had followed cohort-based data to exclusively direct lymph flow toward the ipsilateral axilla? To our knowledge, there are no studies comparing traditional redirecting protocols with newer recommendations for ipsilateral drainage.

There was an error in the original publication. Section 2.5 did not identify the institution where the assessment was made. A correction has been made to Section 2.5, paragraph 1:

In mid-2022, the patient was assessed for a liposuction procedure to reduce excess lymphedema-related tissue in her right arm at the Australian Lymphoedema Education, Research and Treatment Centre (ALERT), Macquarie University. The assessment included clinical examination by a surgical consultant, ICG lymphography, and compression garment evaluation with a specialist physiotherapist (Figure 2). The pre-operative measures showed a 44% greater volume in the right arm compared to the left.

There was an error in the original publication. Section 2.6, paragraph 2 refers to Supplementary Material. The supplementary images should be removed and not replaced:

An analysis of the pre-operative ICG imaging showed a clear lymph pathway from the dorsolateral region of the upper arm, with a large patent radial vessel emptying into the subclavian pathways (Figure 3b).

The authors state that the scientific conclusions are unaffected. This correction was approved by the Academic Editor. The original publication has also been updated.

## Figures and Tables

**Figure 1:**
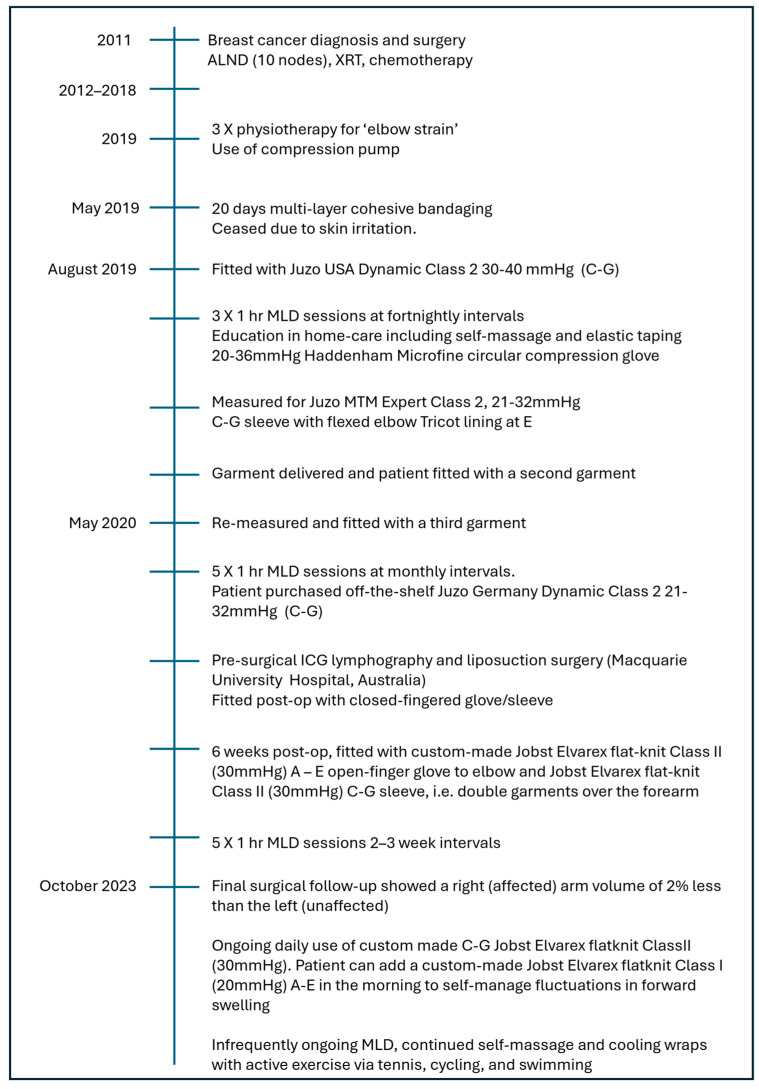
Timeline of breast cancer-related lymphedema diagnosis and treatment. MLD protocol followed redirection protocols according to Vodder. ALND = axillary lymph node dissection; MLD = manual lymph drainage; ICG = indocyanine green.

**Figure 3:**
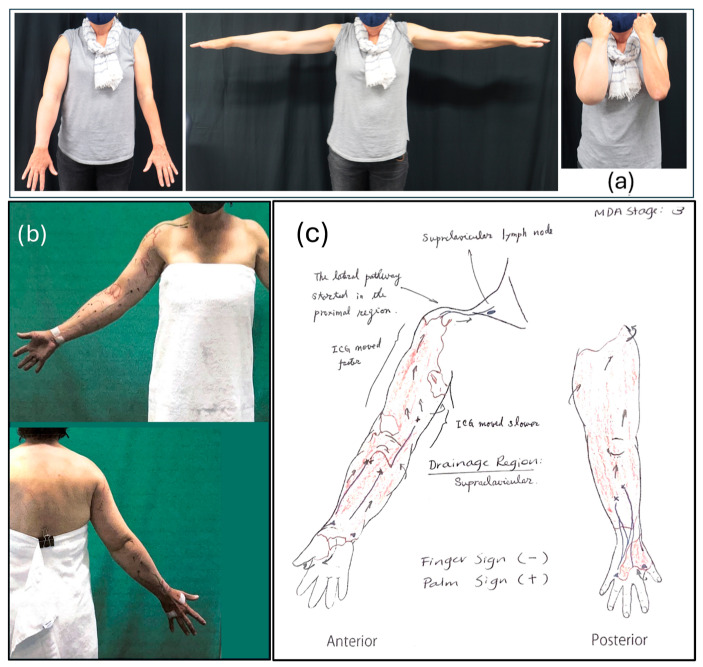
Pre-operative imaging showing (**a**) arm swelling; (**b**) pre-surgical mark-up, with blue lines showing vessels; (**c**) hand-drawn anterior and posterior views of the arm showing areas of dermal back flow (shaded red) and lymph drainage pathways as detected by ICG lymphography (arrows). Photographs and ICG image courtesy the Australian Lymphoedema Education and Research Centre (ALERT) at Macquarie University (with written permission).
