# Supplementary material for: ICG Lymphography Confirms the Presence of an Alternative Lymph Drainage Pathway Following Long-Term Manual Therapy: A Case for Preserving Traditional MLD Approaches
Source: Reports (MDPI). 2025 May 6;8(2):63. doi: 10.3390/reports8020063 (PMC12196954; doi:10.3390/reports8020063)
Supplement: Supplementary file 1 [file reports-08-00063-s001.zip › reports-3588514-supplementary.pdf]

☒ Hand ☐ Foot

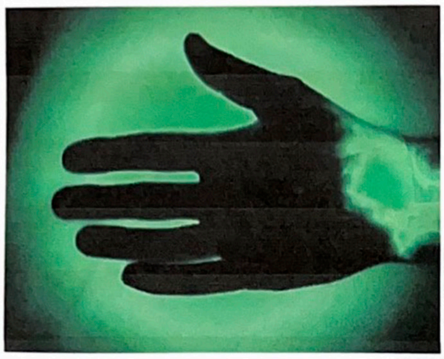

☒ Forearm ☐ Lower leg

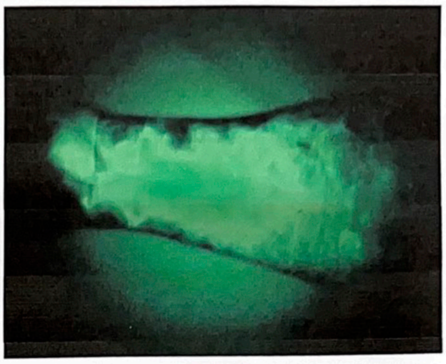

☒ Upper Arm ☐ Thigh

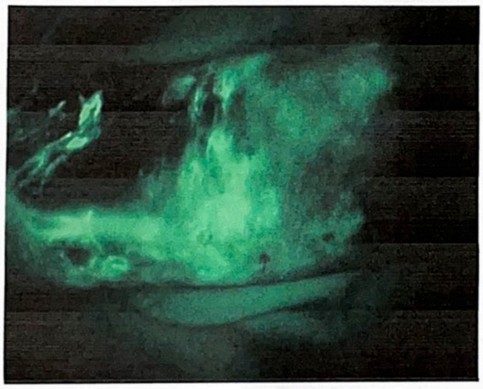

☒ Other: Supraclavicular

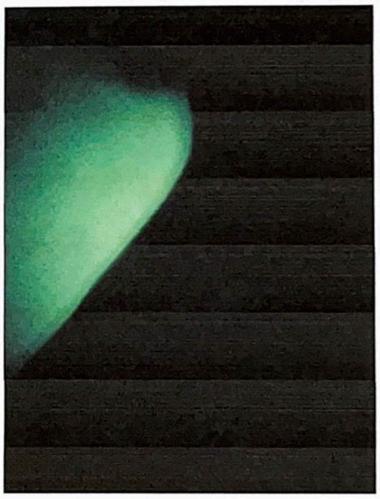

Understanding the ICG Report

To assist you in understanding this report we will explain both the common terminology used and what the colours on your ICG chart represent:

Terminology:

Anterior: the front of the tissue or limb (e.g. the front of the arm, the palm of the hand)

Posterior: the back of the tissue or limb (e.g. the back of the arm or hand)

Medial: the inner part of the tissue or limb (e.g. the inner part of the arm)

Lateral: the outer part of the tissue or limb (e.g. the outer part of the arm)

Lymphatic vessels: vessels that drain/transport lymphatic fluid from the tissue to drainage regions or lymph nodes

Dermal backflow/Congestion: a diagnostic sign of lymphoedema. When the lymphatic vessels are damaged or obstructed, they can no longer drain/transport the lymph fluid. As a result, the lymph fluid flows back up to very small lymphatic capillary vessels within the skin. The body uses these smaller capillary vessels as a compensatory way in which to drain/transport the lymph fluid.

Drainage regions: the region or lymph nodes that have been observed to drain the affected area

Finger/Toe sign: a positive sign indicates that we have seen ICG dye move from the injection sites into the fingers or toes. This is only seen in some people who have lymphoedema and suggests that there may be some swelling of the fingers or toes.

Palm/Sole sign: a positive sign indicates that we have seen ICG dye move from the injection sites to the palm of the hand or the sole of the foot. This sign is only observed in some people with lymphoedema and may influence the direction in which we apply manual lymphatic drainage massage.

MDA stage: an ICG staging system used to indicate the severity of lymphoedema. Lymphoedema severity increases from **stage 0** (normal lymphatic vessels) to **stage 5** (severe lymphoedema).

Figure S1: ICG Lymphography images and report.
